# Shh Signaling from the Injured Lung Microenvironment Drives BMSCs Differentiation into Alveolar Type II Cells for Acute Lung Injury Treatment in Mice

**DOI:** 10.1155/2024/1823163

**Published:** 2024-09-28

**Authors:** Mengyu Wu, Jing Liu, Shu Zhang, Yi Jian, Ling Guo, Huacai Zhang, Junwei Mi, Guoxin Qu, Yaojun Liu, Chu Gao, Qingli Cai, Dalin Wen, Di Liu, Jianhui Sun, Jianxin Jiang, Hong Huang

**Affiliations:** ^1^ Department of Trauma Medical Center Daping Hospital State Key Laboratory of Trauma and Chemical Poisoning Army Medical University, Chongqing 400042, China; ^2^ College of Bioengineering Chongqing University, Chongqing 400044, China; ^3^ Department of Orthopedic Surgery The First Affiliated Hospital of Hainan Medical University, Haikou 570100, Hainan Province, China

## Abstract

Alveolar type II (AT2) cells are key effector cells for repairing damaged lungs. Direct differentiation into AT2 cells from bone marrow mesenchymal stem cells (BMSCs) is a promising approach to treating acute lung injury (ALI). The mechanisms of BMSC differentiation into AT2 cells have not been determined. The Sonic Hedgehog (Shh) pathway is involved in regulating multiple differentiation of MSCs. However, the role of the Shh pathway in mediating the differentiation of BMSCs into AT2 cells remains to be explored. The results showed that BMSCs significantly ameliorated lung injury and improved pulmonary function in mice with ALI. These improvements were accompanied by a relatively high proportion of BMSCs differentiate into AT2 cells and an increase in the total number of AT2 cells in the lungs. Lung tissue extracts from mice with ALI (ALITEs) were used to mimic the injured lung microenvironment. The addition of ALITEs significantly improved the differentiation efficiency of BMSCs into AT2 cells along with activation of the Shh pathway. The inhibition of the Shh pathway not only reduced the differentiation rate of BMSCs but also failed to mitigate lung injury and regenerate AT2 cells. The results confirmed that promoting AT2 cell regeneration through the differentiation of BMSCs into AT2 cells is one of the important therapeutic mechanisms for the treatment of ALI with BMSCs. This differentiation process is highly dependent on Shh pathway activation in BMSCs in the injured lung microenvironment.

## 1. Introduction

Acute respiratory distress syndrome (ARDS) is the most severe manifestation of acute lung injury (ALI) and is a life-threatening condition characterized by acute progressive hypoxic respiratory failure. ALI/ARDS can result from direct lung injuries, such as pneumonia and aspiration, as well as indirect impacts, such as sepsis [1, 2]. Additionally, a study reported that 30% of trauma patients developed ALI/ARDS, resulting in a threefold increase in mortality [3]. Despite major advancements in intensive care research and various therapeutic trials in the past few decades, mortality rates between 35% and 46% have been reported for patients with ALI/ARDS [2, 4]. Currently, there are no drugs available to reduce the mortality of ALI/ARDS [2, 5]. Moreover, 50%–70% of ARDS survivors experience poor quality of life due to persistent limitations in exercise ability and psychological symptoms [5, 6]. Therefore, more specific and effective therapeutic approaches are urgently needed to decrease the morbidity and mortality of ALI/ARDS, which can result in severe hypoxemia.

Damage to the alveolar epithelium is a characteristic feature of ALI/ARDS and causes increased epithelial permeability to proteins and the accumulation of edema fluid in pulmonary septa and alveoli, ultimately leading to hypoxemia [7]. The degree of alveolar epithelial cell damage, particularly alveolar type II (AT2) cell damage, directly determines the extent of both lung function impairment and the prognosis of ALI/ARDS patients [3, 7, 8]. AT2 cells are a major lung epithelial cell type that maintains homeostasis in the alveolar region of the lung by secreting pulmonary surfactant proteins (SPs) to control the surface tension within alveoli. More importantly, AT2 cells serve as alveolar stem cells that can self-renew and differentiate into AT1 cells for re-epithelization of injured alveoli [8]. This approach is beneficial for reconstructing the alveolar-capillary (blood-air) barrier and improving lung function in ALI patients. Therefore, the regeneration of damaged alveolar epithelial cells is a crucial step in the effective treatment of ALI/ARDS [5, 9].

Mesenchymal stem cell (MSC) therapies are emerging as promising therapeutic interventions for patients with ALI/ARDS due to their unique biological properties, including self-renewal [10], anti-inflammatory [11, 12], antimicrobial [13], antifibrotic [14], antioxidative stress [15], paracrine [10], antiapoptotic [15], multiple differentiation [10], and mitochondrial transfer effects [16], which may be of benefit in simultaneously targeting the complex pathogenesis of ALI/ARDS [6, 7, 8, 17, 18]. Preclinical studies and clinical trials have demonstrated the potential efficacy and safety of MSCs administration in the treatment of ALI/ARDS [7, 17]. Recently, MSCs have also been administered in Phase I and II clinical trials in patients with moderate to severe ARDS or sepsis without resulting in any significant side effects or adverse events [18, 19, 20]. Both preclinical and clinical trials have confirmed that ALI/ARDS treatment with MSCs derived from bone marrow, the umbilical cord, or adipose tissue reduces the severity of ALI/ARDS from infectious and noninfectious causes without adverse hemodynamic effects or respiratory compromise [7, 18, 19, 20]. Furthermore, MSCs are multipotent stem cells that can differentiate into alveolar epithelial cells, thereby directly replacing damaged AT2 cells and repairing damaged alveolar structures [8, 21]. However, the efficiency of MSC differentiation into the alveolar epithelium within injured lungs is relatively low, which limits the therapeutic efficacy of MSCs in ALI/ARDS [1, 21]. In fact, the AT2 differentiation rate of MSCs within the damaged lung (ALI) microenvironment has not been determined. Therefore, it is necessary to explore the efficiency of differentiating MSCs into AT2 cells and the associated differentiation mechanism in the microenvironment of ALI/ARDS, as these findings are highly important for improving the therapeutic potential of MSCs. Sonic Hedgehog (Shh), a critical morphogen involved in embryonic lung development, regulates the interaction between epithelial and mesenchymal cell populations in the airway and alveolar compartments. Shh signaling is crucial for alveolar structure formation within lung tissue. Increasing evidence has shown that the expression of the Shh protein is elevated in adult lung diseases such as pulmonary fibrosis, asthma, and chronic obstructive pulmonary disease [22, 23]. However, the expression profile of the Shh protein in ALI/ARDS lung tissues remains unclear. Studies have confirmed that MSCs differentiation into AT2 cells is highly dependent on the injured lung microenvironment [1, 21]. However, the role of the Shh pathway in mediating cross-lineage MSCs differentiation into AT2 cells has not yet been reported, although Shh is critical for the trilineage differentiation of MSCs [24, 25, 26, 27]. Interestingly, the Wnt3a protein can promote the differentiation of MSCs into AT2 cells by activating the canonical Wnt/*β*-catenin pathway [28]. Notably, Wnt3a is an important gene product activated by the Shh pathway [26]. This study investigated the role of MSCs in differentiating into AT2 cells in mouse ALI/ARDS, demonstrated the ability of the injured lung microenvironment to promote the differentiation of MSCs into AT2 cells in vitro and in vivo, and revealed the regulatory role of the Shh signaling pathway in the differentiation of MSCs. These findings might provide new insights and strategies for MSC transplantation therapy in ALI/ARDS patients.

## 2. Materials and Methods

### 2.1. Animals and Ethics Statement

Male mice (C57BL/6, 8–10 weeks, 21–23 g) were provided by the Animal Breeding Center of the Third Military Medical University. These mice were housed under a standard 12-hr light/dark cycle at a constant temperature of 25°C with free access to food and water throughout the experiment. All procedures conducted in this study were performed in adherence to the Guide for the Care and Use of Laboratory Animals (National Institutes of Health Publication No. 86–23, revised 1996). All mouse-related studies were approved by the Ethics Review Committee for Animal Experimentation of our hospital. Primary bone marrow mesenchymal stem cells (BMSCs) were isolated, cultivated, and identified as previously described [21, 29]. BMSCs harvested after 3–7 passages were used for follow-up experiments.

### 2.2. Isolation and Characterization of BMSCs

Primary BMSCs were isolated, cultivated, and identified as previously described [21, 29]. Briefly, BMSCs were harvested by flushing the tibiae and femurs of 1-week-old mice with DMEM/F12 (HyClone, USA) with 10% (vol/vol) fetal bovine serum (FBS; Gibco, USA), 100 *μ*g/mL penicillin, and 100 *μ*g/mL streptomycin; the cells were subsequently cultured in 25-cm^2^ tissue culture flasks in a humidified atmosphere with 5% CO_2_ at 37°C. After 24 hr, the nonadherent cells were removed, and a fresh culture medium was added. The BMSCs exhibited clone-like growth and were passaged at 80% confluence after being digested with TrypLE TM Express (Gibco). BMSCs harvested between 3 and 7 passages were used for follow-up experiments.

### 2.3. Experimental Design

**Figure 1:**
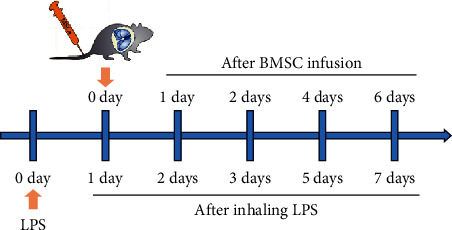
Schematic flowchart and timeline of the study design. A mouse ALI model was established through one-time ultrasonic nebulization inhalation of LPS. (*E. coli* O55:B5). The control mice inhaled sterile physiological saline for 30 min using ultrasonic nebulization. After LPS-induced ALI for 24 hr, the mice further received DiI-labeled BMSCs (0.5 × 10^6^ cells in 100 *μ*L of PBS) with (the GDC group) or without (the BMSC group) pretreatment with the Shh pathway inhibitor GDC-0449 via the tail vein, and the mice in the LPS group received PBS (100 *μ*L).

A solution of lipopolysaccharides (LPS, *Escherichia coli* O55 : B5, L4005; Sigma) was diluted with physiological saline (5 mg/mL). An ALI mouse model was established through one-time ultrasonic nebulization inhalation of LPS (5 mg/mL) for 30 min. After 24 hr, the mice with ALI were injected via the tail vein with a suspension of 100 *μ*L of PBS (the LPS group), 0.5 × 10^6^ BMSCs in 100 *μ*L of PBS (the BMSC group) or 0.5 × 10^6^ GDC-0449-pretreated BMSCs (GDC-0449, an inhibitor of the Shh pathway, pretreated for 4 hr before injection) in 100 *μ*L of PBS (the GDC group) (Figure 1). Prior to injection, BMSCs (DiI-labeled BMSCs) were prelabeled with chloromethyl-1,1-dioctadecyl-3,3,3,3-tetramethylindocarbocyanine perchlorate (CM-DiI, Sigma). The mice were randomly assigned to four groups. There are six animals in the normal control group and 12 mice in each of the BMSC, GDC, and LPS groups. There are three animals at each time point (*n* = 3). BMSCs were incubated with 10 *µ*M DiI for 20 min at 37°C and then centrifuged and resuspended in PBS. The normal mice (the control group) inhaled sterile physiological saline only for 30 min through ultrasonic nebulization.

Following anesthesia, the mice were sacrificed at 1, 2, 4, and 6 days after BMSC infusion using 2% pentobarbital sodium (60 mg/kg) (Figure 1). The left lung samples were immediately collected and fixed in 4% paraformaldehyde (PFA) for pathological analysis, and the right lungs were immediately stored at −80°C for protein analysis via Western blot.

### 2.4. Histological Analysis

Gross images and histological assessment of the injured lung were obtained 1 day after BMSC treatment (equivalent to 2 days post-LPS inhalation). Mice were sacrificed at 1, 2, 4, and 6 days after BMSC infusion. The left lung tissues were fixed in 4% PFA for 6 hr and then preserved in 20% sucrose at 4°C (previously described cryosections for immunofluorescence staining) or fixed in 4% PFA for 12 hr and embedded in paraffin for hematoxylin and eosin (H&E) staining. The right lung tissues were snap-frozen in liquid nitrogen and stored at −80°C until protein analysis via Western blot. Samples were taken from at least three animals at each experimental time point.

The lung histologic analysis was performed by independent pathologists who were blinded to the experimental groups. Lung injury was then evaluated under a light microscope (KF-PRO-003, Konfoong Biotech International Corporation, China). Total lung injury (or histological score) was scored as previously described [30]. The histological score was analyzed based on seven randomly selected high-power fields (hpfs, ×200) in each section according to alveolar and interstitial congestion, hemorrhage, alveolar wall thickness (edema), alveolar and interstitial inflammation with infiltration or aggregation of neutrophils, bullae, and atelectasis; the results were graded on a 0- to 4-point scale: 0, no injury; 1, 25% injury; 2, 50% injury; 3, 75% injury; and 4, 100% injury (or throughout the entire field). The average score of seven hpfs per sample was calculated as the injury score of that sample.

### 2.5. Lung Tissue Extracts

For mimicking the injured lung microenvironment, lung tissue extracts were collected from ALI lung tissues (on the second-day post-LPS injury) and from normal mice. Lung tissues were cut into small pieces and homogenized in ice-cold PBS containing 2 *μ*g/mL protease inhibitors (Solarbio, A8260) at a 1 g : 10 mL PBS ratio. Then, the homogenate was vortexed for 40 s and centrifuged at 12,000 rpm for 10 min to remove debris. The protein concentration was determined using a BCA kit (Beyotime, P0010). The ALI lung tissue extract (ALITE) and normal lung tissue extract (NLTE) were stored at −80°C and used as mimics of the lung microenvironment for in vitro BMSC induction experiments.

### 2.6. In Vitro Differentiation of BMSCs

Primary BMSCs were cultured in DMEM/F12 supplemented with 10% FBS. Cell passaging was performed at 90% confluence. BMSCs were seeded into six-well culture plates at densities of 1 × 10^4^ and 2 × 10^6^ cells per well for pathology and protein analysis, respectively, and were randomly divided into four groups. For analysis of BMSC differentiation into alveolar epithelial cells, BMSCs were cultured under three differentiation conditions, namely, modified small airway growth medium (SAGM, the SAGM group), NLTE (50 *μ*g/mL) + SAGM (the NLTE group), and ALITE (50 *μ*g/mL) + SAGM (the ALI group), for 5 or 10 days. BMSCs in the control group were cultured in DMEM/F12 complete medium alone. The modified SAGM, a medium specially designed for the growth and maintenance of mature distal lung epithelial cells, was composed of basal medium (DMEM/F12; Gibco, USA) in addition to media such as 4% FBS, 1% penicillin-streptomycin solution (Beyotime, China), 1% ITS-G (100×; Gibco, USA), 0.4% NaHCO_3_ (0.9 M), 1.5% HEPES (1 M; Solarbio, China) and 1% L-glutamine(200 mM; Solarbio, China).

For the Shh pathway blockade experiment, BMSCs were seeded into six-well plates at a density of 2 × 10^5^ cells/well. BMSCs were cultured under four differentiation conditions, namely, SAGM + NLTE (50 *μ*g/mL) or SAMG + ALITE (50 *μ*g/mL), with or without 60 *μ*M vismodegib pretreatment for 4 hr (GDC-0449, Selleck, USA). GDC-0449 (GDC) is an inhibitor of the Shh signaling pathway that blocks the activity of the cell surface receptor smoothened (SMO).

Images were captured after BMSC differentiation was induced in vitro for 5 or 10 days. The cells were then harvested for immunofluorescence staining and protein analysis.

### 2.7. Immunofluorescence Staining

Cells and tissue samples (the cryosections of the left lungs and the differentiated BMSCs in vitro) were fixed in 4% PFA for 15 min and 12 hr, respectively. The cell or tissue samples were incubated with the following primary antibodies: anti-prosurfactant protein C (SPC; 1 : 500; Abcam) and anti-podoplanin (T1*α*) (1 : 500; GeneTex). The antibodies were incubated overnight at 4°C after incubation in a blocking solution (5% normal goat serum and 0.25% Triton X-100 in PBS) for 1 hr at room temperature (RT). After the cells were washed with PBS, they were incubated with fluorochrome-conjugated secondary antibodies (1 : 500; Abcam) for 1 hr at 37°C. Nuclei were counterstained with 1 *μ*g/mL 4′, 6-diamino-2-phenylindole (DAPI, Sigma) in PBS. The slides were covered with Immu-Mount (Thermo Fisher Scientific; Waltham, MA, USA). Finally, all the slides were analyzed using an IX71 fluorescence microscope (Olympus, Tokyo, Japan), a panoramic digital pathology scanning microscope (SLIDEVIEW VS2000, Olympus Corporation; Japan), and a confocal laser scanning microscope (STELLARIS 5, Lecia; Germany). For lung tissue sections, the differentiation rate of BMSCs into AT2 cells was determined by determining the ratio of SPC/DiI double-positive cells (SPC + DiI+) to DiI-positive cells (DiI + BMSCs) from the total number of cells at 7 hpfs per sample. For in vitro induction experiments, the differentiation rate of BMSCs into AT2, AT1, or AT1/AT2 (intermediate cell) cells was determined by the ratio of SPC-positive cells (SPC+), T1*α*-positive cells (T1*α*+), and SPC/T1*α* (SPC+/T1*α*+) double-positive cells to DAPI-positive cells (DAPI+) by counting the total number of cells within 7 hpfs per sample.

### 2.8. Lung Function Measurement

Lung function indices of mice in the normal control group, the LPS group, and the BMSC group were measured at 1, 2, 4, and 6 days after BMSC infusion. These assessments were conducted using an animal whole-body volumetric tracer/pulmonary function testing system while the mice were in a conscious and unrestricted state (DSI/BUXCO, WBP, USA). The pulmonary function parameters were measured according to a preset procedure.

### 2.9. Western Blot Analysis

Total protein lysates of cultured cells and lung tissues were prepared using RIPA lysis buffer (Beyotime Biotechnology, Shanghai, China), and the protein concentrations were determined using an ABC Protein Assay Kit (Keygen, Nanjing, China). Equal volumes of protein (50 *µ*g) from each sample were separated via SDS–PAGE (12%–20%) and transferred to PVDF membranes (Millipore, Billerica, MA). These membranes were blocked with 5% nonfat dry milk in Tris-buffered saline for 1 hr at RT and incubated with the following primary antibodies: SPC (1 : 1,000, Abcam), T1*α* (1 : 1000, GeneTex), AQP5 (1 : 1000, Abcam), Shh and *β*-catenin (1 : 800, Santa Cruz), Wnt3a (1 : 800, Proteintech), GLI1 (1 : 800, Affinity), and *β*-actin or GAPDH (1 : 4,000, Proteintech) overnight at 4°C. Following this incubation, the membranes were sequentially incubated with HRP-conjugated secondary antibodies for 2 hr at RT. Finally, the bands were analyzed using a ChemiDoc Touch Imaging System (Bio-Rad, USA). The band density was analyzed using LabWorks 4.6 analysis software.

### 2.10. Statistical Analysis

The data were obtained from at least three independent experiments and analyzed using SPSS 13.0 software. Bivariate analyses (*t*-test and ANOVA) were used to compare the data between groups. A value of *p* < 0.05 was considered to indicate statistical significance.

## 3. Results

### 3.1. BMSC Transplantation for ALI-Alleviated Lung Injury and Improved Lung Function by Promoting AT2 Cell Regeneration

**Figure 2:**
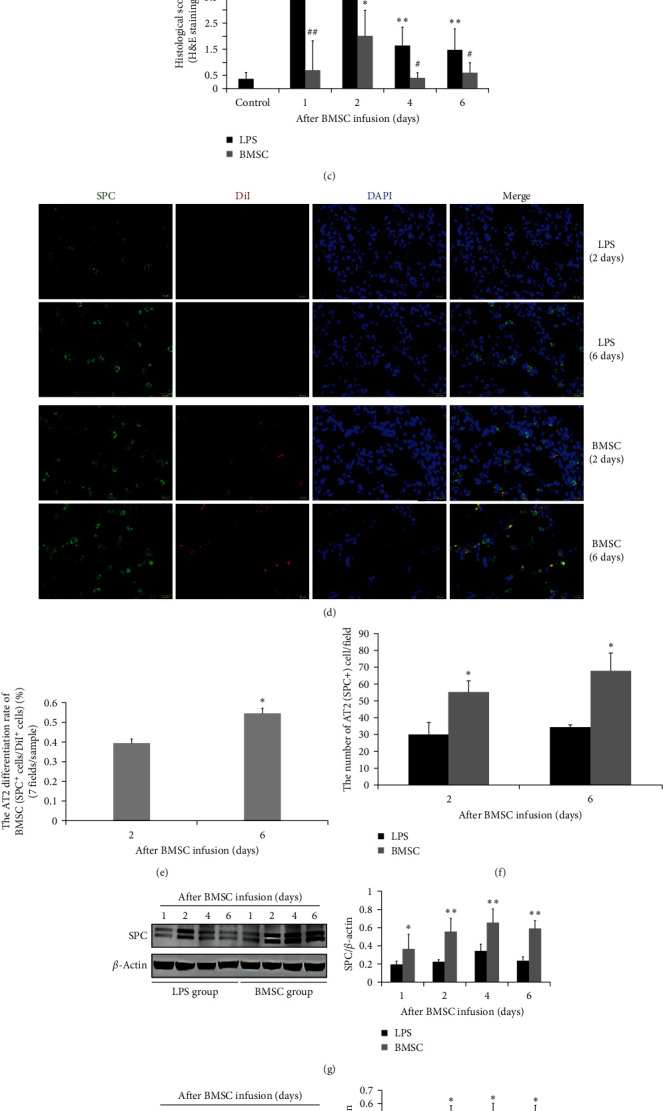
BMSCs improve pathological changes in ALI and promote the differentiation of BMSCs into AT2 cells. The ALI mouse model was established through ultrasonic nebulization inhalation of LPS (5 mg/mL). After 24 hr of ALI induction, either PBS or DiI-labeled BMSCs were injected into the tail vein of the mice. (a) Gross pathological observation of the lungs of the control, LPS-treated, and BMSC groups. (b) H&E staining of lung tissues from the control, LPS-treated, and BMSC groups at 1, 4, and 6 days after BMSC infusion. Scale bar, 100 *μ*m (original magnification: ×200). (c) Evaluation of the degree of lung injury using histopathological score criteria [22]. The data are presented as the mean ± SEM (*n* = 4);  ^*∗*^*p* < 0.05,  ^*∗∗*^*p* < 0.01 vs. the control group; ^#^*p* < 0.05, ^##^*p* < 0.01 vs. the LPS group. (d) Representative immunofluorescence images of SPC-stained lung tissues from the LPS and BMSC groups at 2 and 6 days after BMSC infusion. Immunofluorescence labeling of DiI-BMSCs (DiI, red) and AT2 cells (SPC, green) and the nuclear stain DAPI (blue). BMSCs that differentiated into AT2 cells (double-positive for DiI and SPC) appeared as yellow or brown cells. Scale bar, 25 *µ*m. (e) The differentiation rate of BMSCs into AT2 cells in each lung sample in the BMSC groups was determined by calculating the ratio of the total number of double-positive cells (DiI + SPC+) to the total number of DiI-positive cells at seven randomly selected hpfs (×200) at 2 and 6 days after BMSC infusion. The data are presented as the mean ± SEM (*n* = 3).  ^*∗*^*p* < 0.05, vs. the second day in the BMSC groups. (f) The number of AT2 cells in each lung sample in the LPS and BMSC groups was assessed by calculating the average number of SPC-positive cells (SPC+) at 7 hpfs per section (×400), indirectly reflecting the regeneration of AT2 cells in the lungs. The data are presented as the mean ± SEM (*n* = 3).  ^*∗*^*p* < 0.05 vs. the LPS groups. (g) Western blot analysis of the expression of SPC. (h) Western blot analysis of the expression of AQP5 in lung tissues from the LPS and BMSC groups. The data are presented as the mean ± SEM (*n* = 3).  ^*∗*^*p* < 0.05,  ^*∗∗*^*p* < 0.01 vs. the LPS group.

To elucidate the effect of MSC differentiation into AT2 cells during MSC treatment of ALI, we established a mouse model of ALI induced by LPS through one-time ultrasonic nebulization. Twenty-four hours after ALI induction, DiI-labeled BMSCs were injected via the tail vein. Gross observation revealed that the lung surface of the normal mice was pink without congestion or hemorrhagic lesions, while the lungs of the LPS-treated mice were dark red due to diffuse pulmonary congestion and hemorrhage. However, the lung surfaces of the BMSC group were pink in color, similar to those of normal lungs, at 4 and 6 days post-BMSC infusion, with no apparent congestion or hemorrhage in the lungs, except for mild congestion on the first day (Figure 2(a)). Similarly, H&E staining revealed that the alveoli in the normal control group exhibited a uniform and intact morphology; thin alveolar walls with no infiltration or exudation of inflammatory cells in the alveoli, and congestion or edema of the alveoli and pulmonary interstitium (Figure 2(b)). However, the lungs of the mice in the LPS group exhibited the disappearance of the normal lung tissue structure, accompanied by interstitial congestion, infiltration, and exudation of a large number of inflammatory cells into the alveolar spaces, thickening of the alveolar walls, local formation of bullae and atelectasis (collapsed alveolar cavity), and pulmonary hemorrhage (presence of red blood cells in the alveolar space). These pathological changes in the LPS group were most severe on the second day after LPS injury (ALI) but were somewhat alleviated on the seventh day. Transplantation of BMSCs significantly alleviated the pathological damage induced by LPS at each time point. On the sixth day after BMSC treatment (relative to the seventh day after ALI), the lung tissue structure was almost completely restored to a normal lung state. The histological score at various time points was significantly greater in the LPS group than in the normal control group and BMSC group, with the exception of the second day after BMSC infusion (Figure 2(c)). In the BMSC group, immunofluorescence staining revealed that some of the implanted DiI-BMSCs (red, DiI + cells) co-expressed the SPC protein at all time points (green, DiI + SPC + double-positive cells) (Figure 2(d)), which indicated that the BMSCs underwent differentiation into AT2 cells. The differentiation rates of BMSCs into AT2 cells (DiI + SPC + /DiI+) on the second and sixth days after BMSC infusion were 39.3% and 54.4%, respectively, and these rates were significantly greater on the sixth day than on the second day (Figure 2(e)). Furthermore, Western blot analysis confirmed that the expression levels of SPC and AQP5 at each time point in the lung tissues of the BMSC group were significantly greater than those in the LPS group (Figures 2(g) and 2(h)). Additionally, the number of SPC + cells per field (the average value of the total number of SPC + cells at 7 hpf) was significantly greater in the BMSC group than in the LPS group (Figures 2(d) and 2(f)). These results suggested that a substantial proportion of DiI-labeled BMSCs (red) implanted in the lungs might differentiate into AT2 cells and that BMSCs have a reparative effect on damaged lungs by promoting an increase in the number of AT2 cells in the treatment of ALI/ARDS.

**Figure 3:**
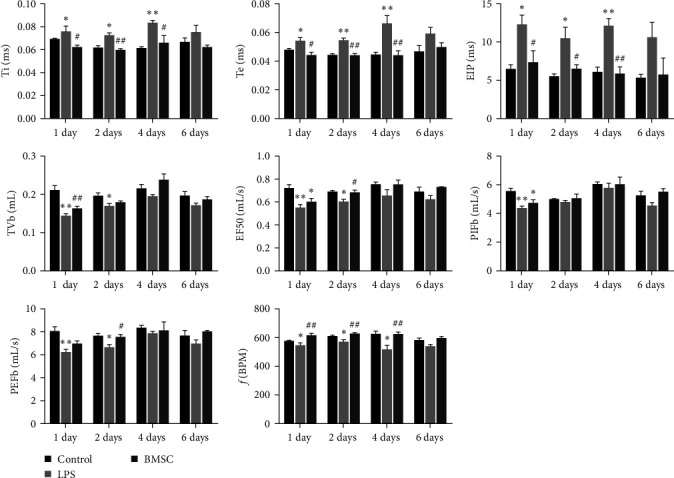
BMSCs improve lung function in LPS-induced ALI. After 24 hr of ALI induction, either PBS or DiI-labeled BMSCs were injected into the tail vein of the mice (*n* = 10).  ^*∗*^*p* < 0.05,  ^*∗∗*^*p* < 0.01, vs. the normal control. ^#^*p* < 0.05, ^##^*p* < 0.01 vs. the LPS group. Ti (ms), inspiratory time; Te (ms), expiratory time; EIP (ms), end-inspiratory pause; TVb (mL), tidal volume; EF50 (mL/s), expiratory flow 50; PIFb (mL/s), peak inspiratory flow; PEFb (mL/s), peak expiratory flow; *f* (BPM), frequency; MVb (mL), minute volume.

Due to the close correlation between pulmonary dysfunction and alveolar structural damage, we observed and compared lung function indices between the LPS group and the BMSC treatment group (Figure 3). The results showed that the general indices (Ti, Te, and EIP) of the LPS group were significantly abnormal compared to those of the control and BMSC groups, while these indices did not significantly differ between the BMSC group and control group. Ti and Te were significantly greater in the LPS group than in the normal group, indicating the presence of incomplete obstruction in the lower airways, such as pulmonary edema and emphysema (i.e., pulmonary bullae); TVb, an index reflecting lung volume, was significantly lower in the LPS group than in the normal group at 1 and 2 days post-BMSC infusion, indicating insufficient ventilation and pulmonary edema. However, this indice in the BMSC group was close to normal level at each time point. The airway obstruction index (EF50) reflects airway resistance and indicates the severity of obstructive ventilation disorders. These indices in the LPS group showed a significant decrease compared to the normal group at 1 and 2 days post-BMSC infusion, but it returned to normal level at 2 days after BMSC treatment. Gas conduction indices (PIFb, PEFb) mainly reflect small airway obstruction and a decrease in their value indicated poor ventilation. Compared with those in the control group, PIFb and PEFb in the LPS group decreased significantly at 1 and 2 days post-BMSC infusion but remained normal in the BMSC group. The lung ventilation indice *f* (BPM) of the LPS group was significantly lower than that of the normal group at 1, 2, and 4 days after BMSC infusion, while *f* in the BMSC group was not significantly different from that of the normal group. On the sixth day post-BMSC infusion, there was no significant difference in all lung function indices among the three groups.

Collectively, the results showed significant abnormalities in various lung function indices in the LPS group, indicating impaired lung function. However, BMSC treatment significantly improved lung function abnormalities in the LPS-induced mice with ALI. A considerable proportion of BMSCs differentiating into AT2 cells contributed to lung repair through regeneration of AT2 cells and subsequent repair of AT1 cells in the lungs (as indicated by the increase in AQP5 expression in the BMSC group), which might be one of the important therapeutic mechanisms of BMSCs in the treatment of ALI/ARDS [31].

### 3.2. Enhanced Differentiation of BMSCs into AT2 Cells in a Mimicked ALI Microenvironment In Vitro

**Figure 4:**
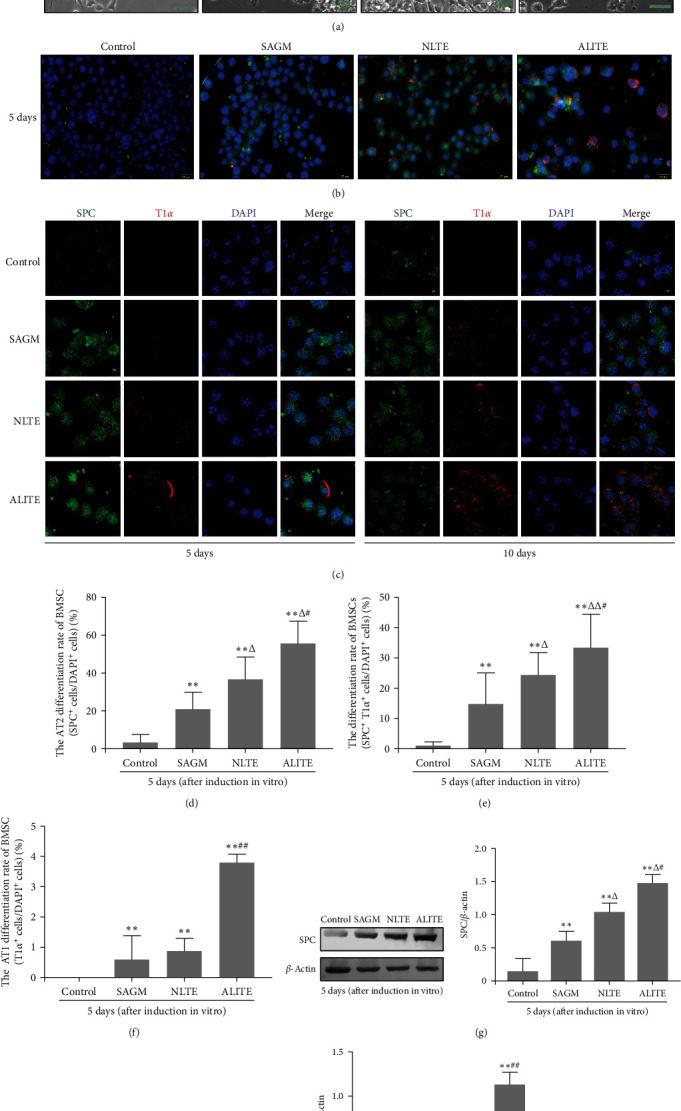
The ALI microenvironment with ALITEs promotes the differentiation of BMSCs into alveolar epithelial cells in vitro. The alveolar epithelial cell differentiation of BMSCs was induced with modified small airway growth medium (SAGM), normal lung tissue extract (NLTE), and ALI lung tissue extract (ALITE) for 5 and 10 days in vitro. BMSCs in the control group were cultured in DMEM/F12 complete medium alone. Morphological changes and the expressions of the lung epithelial cell markers SPC and T1*α* in induced BMSCs were observed and detected. (a) Morphological changes: The morphological changes in the BMSCs were observed under an inverted microscope. Scale bar, 25 *μ*m. (b) Immunofluorescence staining: Immunofluorescence images of induced BMSCs treated with SPC (green) and T1*α* (red) were observed under a fluorescence microscope after 5 days of induction. The nuclei were stained with DAPI (blue). Scale bar, 20 *μ*m. (c) Confocal laser scanning microscope: Immunofluorescence staining images of SPC and T1*α* were observed via confocal microscopy after BMSCs were induced for 5 and 10 days. Scale bar, 10 *μ*m. (d–f) Differentiation ratios: Differentiation ratios of BMSCs into AT2 cells (SPC+), intermediate cells (SPC+ /T1*α* +), and AT1 cells (T1*α* +) after BMSC therapy were assessed after 5 days of induction. The data are presented as the mean ± SEM (*n* = 3). (g and h) SPC and T1*α* protein expressions: Western blot analysis was also conducted to assess the expressions of SPC and T1*α* in induced BMSCs after 5 days. The data are presented as the mean ± SEM (*n* = 3),  ^*∗∗*^*p* < 0.01 vs. the control group; *Δp* < 0.05, *ΔΔp* < 0.01 vs. the SAGM group; ^#^*p* < 0.05, ^##^*p* < 0.01 vs. the NLTE group.

To investigate the impact of the ALI microenvironment on BMSC differentiation into alveolar epithelial cells, particularly AT2 cells, we obtained lung tissue extracts (or homogenates) from normal (NLTEs) and LPS-induced ALI (ALITEs) lung tissues to mimic the pulmonary microenvironment. To observe the effects of these combinations on BMSC differentiation into AT2 cells, we added NLTEs and ALITEs to the in vitro induction culture systems. Under an inverted microscope, we observed that the BMSCs in the normal control group (cultured in DMEM/F12) exhibited short spindles or round shapes. After 5 days of induction in the SAGM, NLTE, and ALITE media, the majority of the BMSCs exhibited no significant morphological changes, except for a few cells that exhibited an epithelial-like morphology. After 10 days of induction, the morphology of many BMSCs in the three induction groups significantly changed, especially in the ALITE group; most of the cells exhibited an epithelial-like morphology characterized by a polygonal shape and tight intercellular junctions (Figure 4(a)).

Markers for alveolar epithelial cells, SPC (specific marker for AT2 cells), and T1*α* (specific for AT1 cells) were used to track BMSC differentiation. Fluorescence microscopy and confocal microscopy revealed that the majority of BMSCs in the SAGM, NLTE, and ALITE induction groups expressed SPC (green fluorescence in the cytoplasm), with some expressing both SPC and T1*α*, and fewer cells expressed only T1*α* (red fluorescence on the cell membrane) after 5 and 10 days of in vitro induction. However, the BMSCs in the control group did not express SPC or T1*α* (Figures 4(b) and 4(c)).

The number of SPC/T1*α* double-positive and T1*α*-positive cells increased significantly in these three induction groups after 5 and 10 days of induction, especially in the ALITE group. After 10 days of induction in vitro, the green fluorescence intensity (SPC) in the cells of the three induction groups exhibited a decreasing trend, while the red fluorescence intensity exhibited an increasing trend compared with that after 5 days of induction. Therefore, T1*α*-positive cells expressing only red fluorescence were the most abundant in the ALITE group after induction for 5 and 10 days (Figure 4(c)). Similarly, the ratios of SPC-positive cells, T1*α*-positive cells, and SPC/T1*α* double-positive cells (intermediate cells) to DAPI-positive cells represented the differentiation ratios of BMSCs into AT2, AT1, and intermediate AT (SPC+/T1*α*+) cells, respectively. The differentiation ratios were significantly greater in the three induction groups (the SAGM, NLTE, and ALITE groups) than in the control group after 5 days (Figures 4(d), 4(e), and 4(f)). These values in both the NLTE and ALITE groups increased markedly compared to those in the SAGM group, and they were significantly greater in the ALITE group than in the NLTE group; thus, these ratios in the ALITE group were the highest among the three induction groups, which was consistent with the findings of fluorescence microscopy and confocal microscopy. Furthermore, Western blot analysis confirmed the elevated expression levels of SPC and T1*α* in these three induction groups compared to those in the control group, with the highest expression in the ALITE group (Figures 4(g) and 4(h)). These results indicated that SAGM, NLTEs, and ALITEs could promote the differentiation of BMSCs into alveolar epithelial cells, particularly AT2 cells, in vitro, with the ALITE (mimicked injured lung microenvironment) group exhibiting the strongest induction effect.

### 3.3. Activation of the Shh Signaling Pathway Promotes the Differentiation of BMSCs into Alveolar Epithelial Cells

**Figure 5:**
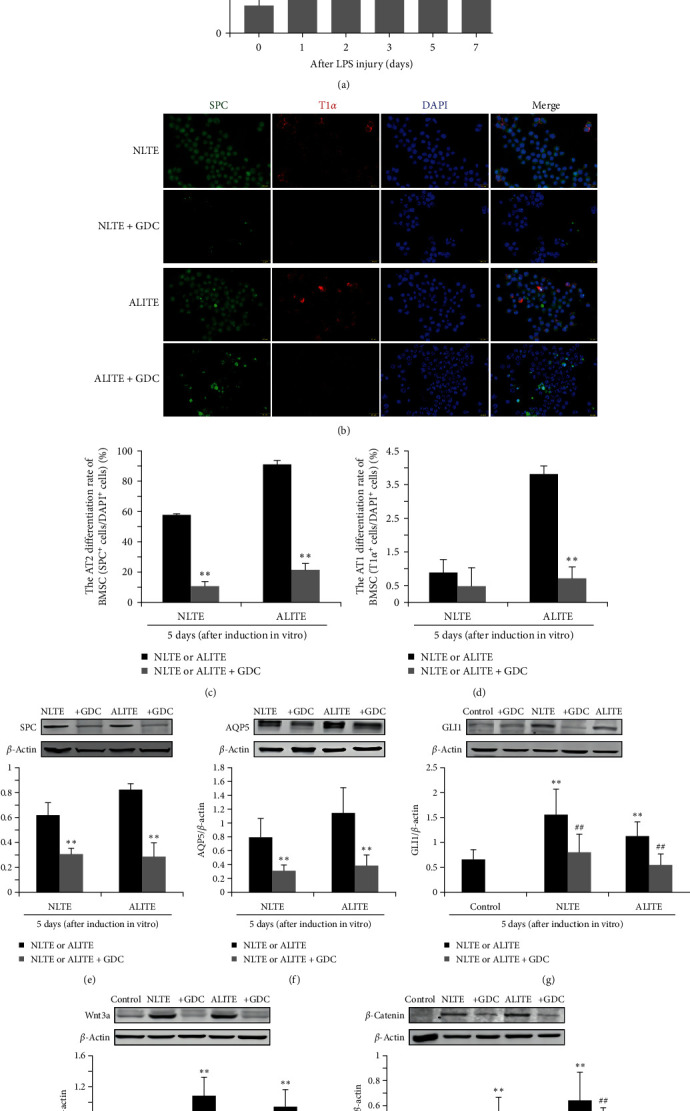
The Shh signaling pathway plays an important role in the differentiation of BMSCs into AT2 cells in the mimicked lung microenvironment of ALI. BMSCs were induced and differentiated for 5 days in NLTE and ALITE induction culture systems, both with or without pretreatment with the Shh pathway inhibitor GDC-0449 (GDC) for 4 hr. The results were as follows: (a) Shh protein expression: Western blot analysis of Shh protein expression in mouse lung tissue after ALI. The data are shown as the mean ± SEM (*n* = 4),  ^*∗∗*^*p* < 0.01 vs. the control group (Day 0). (b) Immunofluorescence staining: Immunofluorescence images of SPC (green) and T1*α* (red) in induced BMSCs were observed under a fluorescence microscope. The nuclei were stained with DAPI (blue). Scale bar, 20 *μ*m. (c and d) Differentiation ratios: Assessment of the differentiation ratios of BMSCs into AT2 cells (SPC+) and AT1 cells (T1*α*+) after BMSCs were induced for 5 days. The data are presented as the mean ± SEM (*n* = 3).  ^*∗∗*^*p* < 0.01 vs. the NLTE or ALITE group. (e and f) Marker expression: Western blot analysis of SPC and AQP5 expression in BMSCs postinduction for 5 days. The data are presented as the mean ± SEM (*n* = 3)  ^*∗∗*^*p* < 0.01 vs. the NLTE or ALITE group. (g–i) Western blot analysis of GLI1, Wnt3a, and *β*-catenin protein expression in BMSCs after induction for 5 days. The values are presented as the mean ± SEM (*n* = 4).  ^*∗∗*^*p* < 0.01 vs. the control group; ^##^*p* < 0.01 vs. the NLTE or ALITE group.

Since the Shh signaling pathway is an important pathway that regulates cell differentiation and morphogenesis, especially stem cell differentiation [26, 28], the role of the Shh pathway in the differentiation of BMSCs into AT2 cells was investigated. The expression pattern of the Shh protein in lung tissues after ALI was assessed. The results showed that the expression level of the Shh protein was very low in normal mouse lung tissue (Day 0, control group). Compared with those in the control group, the protein levels of Shh in injured lung tissues were significantly greater at 1, 2, 3, 5, and 7 days after ALI (Figure 5(a)).

Ultimately, to examine the role of the Shh pathway in BMSC differentiation, we collected lung tissue extracts on the second day after ALI to establish a model of the injured lung microenvironment. NLTEs or ALITEs were added to the induction system after 4 hr of pretreatment with or without 60 *μ*M vismodegib (GDC-0449), a Shh pathway inhibitor. After 5 days of in vitro induction, immunofluorescence staining revealed a significant decrease in BMSC differentiation into AT2 and AT1 cells in the GDC inhibitor groups and in the NLTE + GDC and ALITE + GDC groups compared to the corresponding NLTE and ALTTE groups without the inhibitor (Figures 5(b), 5(c), and 5(d)). This decrease was accompanied by reduced expression of the SPC and AQP5 proteins (Figures 5(e) and 5(f)), as well as the expression of key downstream proteins, including GLI1, Wnt3a, and *β*-catenin in the Shh pathway (Figures 5(g), 5(h), and 5(i)). However, the levels of these downstream target proteins of the Shh pathway in the NLTE and ALITE alone groups were significantly greater than those in the control group (BMSCs cultured in DMEM/F12 medium). These results indicated that the Shh pathway has a profound impact on the differentiation of BMSCs into alveolar epithelial cells. The Shh signal originating from the ALI microenvironment might trigger BMSC differentiation into AT2 cells through the activation of the Shh pathway within BMSCs.

### 3.4. Blocking the Shh Pathway in BMSCs Attenuates Their Therapeutic Potential in Mice with ALI by Reducing the Differentiation Efficiency of BMSCs into AT2 Cells

**Figure 6:**
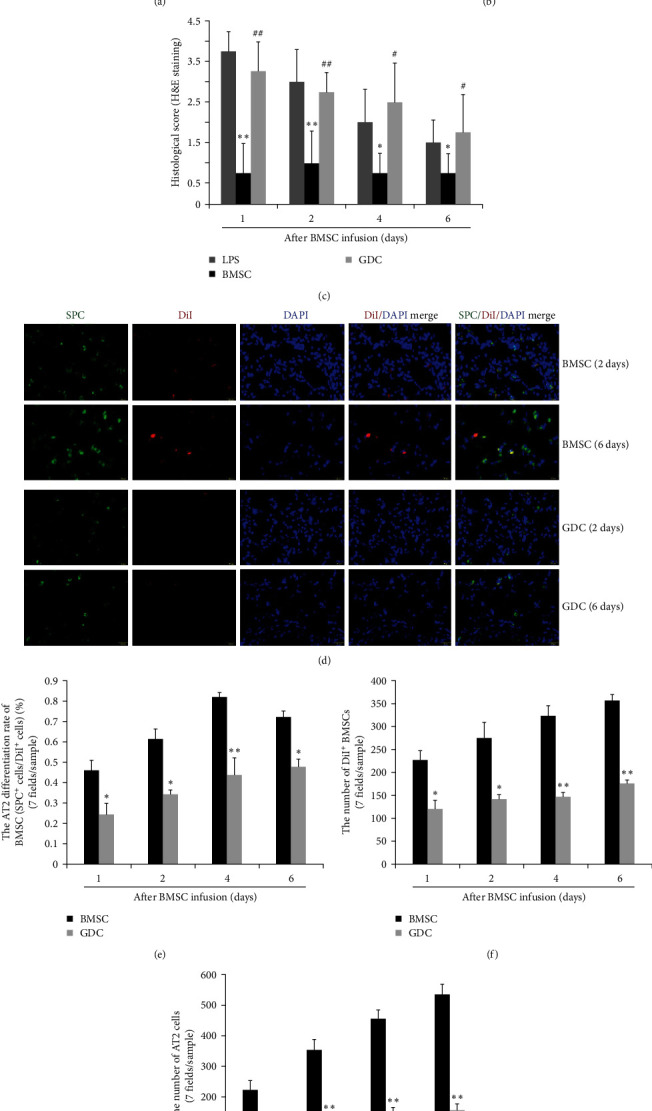
The Shh pathway antagonist GDC-0449 inhibits the regeneration of AT2 cells in ALI lungs after BMSC treatment. A mouse ALI model was established through one-time ultrasonic nebulization inhalation of LPS (5 mg/mL, 30 min). DiI-labeled BMSCs were injected via the mouse tail vein after ALI for 24 hr. Histological analysis of lung tissues was performed at 1, 2, 4, and 6 days after BMSC infusion. (a) Gross pathological observation: Gross pathological observation of lung tissues in the LPS, BMSC, and GDC groups. (b) H&E staining: H&E staining of lung tissues from the LPS, BMSC, and GDC groups. Scale bar, 2.5 mm. (c) Histopathological score: The degree of lung injury was evaluated using histopathological score criteria. The data are shown as the mean ± SEM (*n* = 3);  ^*∗*^*p* < 0.05,  ^*∗∗*^*p* < 0.01 vs. the LPS group; ^#^*p* < 0.05, ^##^*p* < 0.01 vs. the BMSC group. (d) Immunofluorescence staining: Representative immunofluorescence images of lung tissues from the BMSC and GDC groups at 2 and 6 days after BMSC infusion. Immunofluorescence labeling of DiI-BMSCs (DiI, red) and AT2 cells (SPC, green) and nuclear staining with DAPI (blue). BMSCs that differentiated into AT2 cells (double-positive for DiI and SPC) appeared as yellow or brown cells. Scale bar, 25 *µ*m. (e) AT2 differentiation rate: The differentiation rate of BMSCs into AT2 cells in each lung sample from the BMSC and GDC groups at 1, 2, 4, and 6 days after BMSC infusion was determined by calculating the ratio of the total number of double-positive cells (DiI + SPC+) to the total number of DiI-positive cells in seven randomly selected high-power fields (hpfs, ×200). The data are shown as the mean ± SEM (*n* = 3).  ^*∗*^*p* < 0.05,  ^*∗∗*^*p* < 0.01 vs. the BMSC group. (f) Total DiI-positive cells: Histological analysis of the total number of DiI-positive cells (red) in seven randomly selected high-power fields per section at 1, 2, 4, and 6 days after BMSC infusion. The data are shown as the mean ± SEM (*n* = 3).  ^*∗*^*p* < 0.05,  ^*∗∗*^*p* < 0.01 vs. the BMSC group (g) Total SPC-positive cells: Histological analysis of the total number of SPC-positive cells (AT2, green) in seven randomly selected high-power fields (×200) per section at 1, 2, 4, and 6 days after BMSC infusion. The data are shown as the mean ± SEM (*n* = 3).  ^*∗∗*^*p* < 0.01 vs. the BMSC group.

To further confirm the potential impact of the Shh signaling pathway on BMSC differentiation into AT2 cells and its therapeutic efficacy in the treatment of ALI, we generated a mouse model of LPS-induced ALI. On the first day after ALI induction, DiI-labeled BMSCs pretreated with or without the Shh pathway antagonist GDC for 4 hr were injected into the tail vein of mice with ALI. Gross pathological observation revealed that, compared with that in the LPS group, the pathological damage to the lungs in the BMSC group was significantly mitigated at 1, 4, and 6 days after BMSC infusion, as indicated by the reduced pulmonary surface congestion and hemorrhage. By the sixth day, the lung surface appeared pink in color, close to that of the normal lung. Conversely, the gross pathological changes in the lungs of the mice in the GDC group were similar to those in the LPS group at various time points, as the GDC group exhibited extensive congestion and hemorrhage (Figure 6(a)). The histopathological changes in the lung tissues of each group were consistent with the gross pathological observations (Figure 6(b)). Like those in the LPS group, the lung tissue in the GDC group exhibited severe histopathological damage at various time points, characterized by diffuse inflammatory cell infiltration, thickened alveolar walls (interstitial edema), destroyed and disordered alveoli, and collapsed partial alveoli. In contrast, these pathological changes in the lung tissue of the BMSC group were significantly milder. Similarly, the histological injury scores determined via H&E staining further confirmed that the score in the BMSC group was significantly lower than that in the LPS and GDC groups, while there was no difference in histological scores between the LPS and GDC groups (Figure 6(c)). These results indicated that BMSC transplantation significantly reduced lung injury in mice with ALI while blocking the Shh pathway in BMSCs, which might weaken the therapeutic potential of BMSCs. These findings suggested that the Shh pathway plays an important role in the therapeutic potential of BMSCs in the treatment of ALI and that the Shh protein serves as an important mediator of the interaction between transplanted exogenous BMSCs and the injured lung microenvironment.

However, compared with those in the BMSC group, the number of DiI-BMSCs coexpressing SPC in the lung tissue of the GDC group was significantly lower (Figure 6(d)). The differentiation rate of BMSCs into AT2 cells in the GDC group was significantly lower than that in the BMSC group at various time points after BMSC infusion (Figure 6(e)). Additionally, there was a significant decrease in the number of DiI-BMSCs (red) and SPC-positive (green) cells in the lung tissues of the GDC group (Figures 6(f) and 6(g)). Therefore, blocking the Shh pathway in transplanted BMSCs with GDC not only suppressed the differentiation of BMSCs into AT2 cells but also inhibited the regeneration of AT2 cells in the lungs. Finally, these findings seriously undermine the therapeutic potential of BMSCs.

## 4. Discussion

Loss of alveolar type 2 (AT2) cells is the primary pathological basis for the development of ALI/ARDS and leads to impaired gas exchange barrier function and hypoxemia [32, 33]. Given the importance of AT2 cells, promoting the regeneration and efficient repair of injured AT2 cells as early as possible is crucial for improving lung function, preventing the development of ALI/ARDS, and preventing advanced pulmonary fibrosis. Although numerous studies have demonstrated the beneficial effects of MSC-based therapy for ALI/ARDS [6, 7, 8, 17, 19, 20, 21], the efficiency and mechanisms of MSC differentiation in the injured pulmonary microenvironment are poorly understood. The findings of this study shed light on the potential mechanisms underlying the therapeutic effects of BMSCs in the treatment of ALI and ARDS.

In the present study, BMSC treatment significantly reduced lung pathological damage and improved lung function in mice with ALI, which was consistent with the findings of other studies [2, 17, 18, 19, 20, 21]. However, interestingly, during the early stages of ALI treatment, a relatively high proportion of BMSCs differentiated into AT2 cells, and a significant increase in the number of AT2 cells in the lung tissue was observed in the BMSC group on the second and sixth days after BMSC treatment, along with an increase in SPC and AQP5 protein expression at various time points. These results indicated that BMSC therapy could promote the regeneration of lung AT2 cells and the repair of AT1 cells. The repair of AT1 cells is based on the ability of AT2 cells to differentiate into AT1 cells during lung injury [1, 34, 35], as indicated by the increased expression of AQP5 in the lung tissues of the BMSC group. Finally, the injured alveoli were repaired, thereby significantly alleviating pathological lung damage (by decreasing the pathological injury score) and improving lung function in LPS-induced ALI.

In this study, we observed a high proportion of BMSCs differentiating into AT2 cells in the early stages of BMSC treatment for ALI, which was inconsistent with the findings of several studies suggesting a very low efficiency (approximately 5%) of MSC differentiation into alveolar epithelial cells and that the therapeutic effects of MSCs in tissue regeneration and repair are not primarily mediated through differentiation into tissue cells but rather through their paracrine functions [2, 21, 33, 34, 36, 37, 38]. Indeed, previous studies have never reported on the ability of BMSCs differentiate into AT2 cells in ALI lung tissue after BMSC treatment [1, 21, 22]. Given that BMSC engraftment in the lung tended to result in rapid differentiation into AT2 cells with a relatively high differentiation rate (39.3% and 54.4% on the second and sixth days after BMSC infusion, respectively), our findings suggest that BMSCs participate in the repair of injured lungs predominantly by direct differentiation into AT2 cells, facilitating the regeneration of AT2 cells, which might be one of the important therapeutic mechanisms of BMSCs in treating ALI/ARDS. Additionally, these results also suggested that certain factors might be involved in triggering the differentiation of BMSCs into AT2 cells in the lung microenvironment in ALI patients.

The host environment, especially inflammatory active environments, plays a critical role in directing infused MSCs toward different phenotypes with different functions [35]. The differentiation of MSCs is closely related to the microenvironment in which they are located [1, 2, 35]. For further analysis of the impact of the ALI lung microenvironment on BMSC differentiation into AT2 cells, normal and ALI mouse lung tissue extracts (NLTEs and ALITEs) were used as lung microenvironment mimics and added to the alveolar epithelial differentiation induction culture system. Inverted microscopy and immunofluorescence analysis revealed that the longer the induction time was, the greater the number of cells exhibiting epithelial-like morphological changes and the greater the number of T1*α*-positive cells (red fluorescent, AT1 cells), particularly in the ALITE group. Accordingly, the differentiation rate of BMSCs into AT2, AT1, and SPC/T1*α* double-positive cells, as well as the expression levels of SPC and T1*α* protein, were the highest in the ALITE group. Our results on BMSC differentiation into AT1 and AT2 cells using ALITEs were consistent with those reported by Maria et al. [39]. These researchers also found that the proportion of BMSCs differentiating into AT2 cells was significantly greater when cocultured with injured lung tissue cells than when cocultured with normal lung tissue cells. Another study of MLE-12 cells (a mouse lung epithelial cell line) in a Transwell chamber revealed that MLE-12 cells significantly promoted BMSC differentiation into AT2 cells [26]. Our results and those of previous studies suggested that the differentiation of MSCs is strongly dependent on their microenvironment, which has a profound impact on their differentiation direction [2, 28, 39, 40]. These results suggested that the lung microenvironment in ALI/ARDS patients contains strong mediators that are capable of triggering the differentiation of BMSCs into alveolar epithelial cells.

The secreted protein Shh is a morphogen that plays a critical role in mammalian embryonic development by mediating essential tissue morphogenesis events [23, 24, 25, 26]. GLI1 is both a transcription factor and a target of Shh signaling and reliably reports Shh pathway activity [41]. This pathway is typically silent in terminally differentiated cells but can be reactivated in response to tissue injury to mediate cell regeneration and repair [23, 24, 42, 43, 44]. The results showed that the expression of the Shh protein was very low in the normal mouse lung, but it was strongly expressed in the lungs from Day 1 to Day 7 after ALI. This finding suggested a persistently high level of the Shh protein (Shh signaling) in lung tissue (in ALITE) during early ALI.

Shh signaling also maintains adult stem cells in various fully developed tissues and is involves in regulating the multilineage differentiation processes of MSCs, such as osteogenesis [45, 46], chondrogenesis, and adipogenesis [25, 27, 28]. In this study, both NLTEs and ALITEs (both containing the Shh protein) significantly promoted the differentiation of BMSCs into AT2 and AT1 cells in vitro. However, the efficiency of ALITEs in promoting BMSC differentiation into the alveolar epithelium was significantly greater than that of NLTEs. The application of the Shh pathway inhibitor vismodegib (GDC-0449) effectively weakened the induction effects of ALITEs and NLTEs, resulting in a significant decrease in the differentiation rate of AT2 and AT1 cells, as well as in the expression levels of SPC and T1*α*. These results suggested that Shh signaling at least partially mediated the effect of the injured lung microenvironment on the differentiation of BMSCs into the alveolar epithelium.

**Figure 7:**
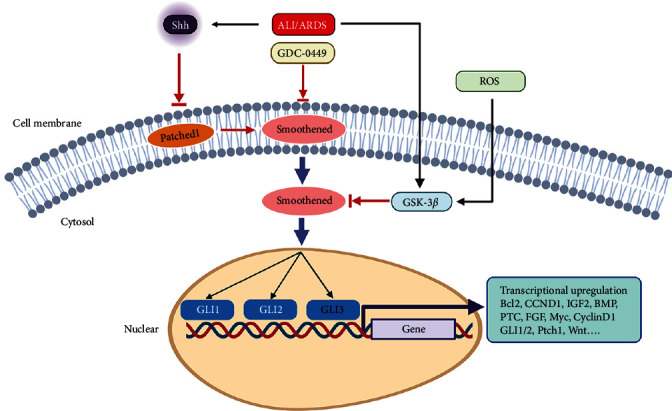
Schematic representation of the activation of the Shh pathway in implanted BMSCs in the microenvironment of ALI/ARDS. The highly expressed Shh protein in injured lung tissue binds to the Patched1 receptor on the surface of transplanted BMSCs and activates the Shh signaling pathway in BMSCs. Once Shh binds to Patched1, the inhibitory effect of Patched1 on the Smoothed (SMO) receptor is relieved. SMO activates the transcription factors GLI1 and GLI2 through transduction of Shh signals. Subsequently, GLI transcription factors translocate into the nucleus, where they recognize GLI binding sites in DNA and stimulate the transcription of target genes, including Bcl-2, IGF2, FGF, BMP, Myc, cyclin D1, GLI1/2 itself, and Wnt, which are closely related to BMSC differentiation. ROS: reactive oxygen species.

Previous studies have demonstrated that activation of the Wnt/*β*-catenin signaling pathway promotes the differentiation of MSCs into AT2 cells [21]. Wnt proteins are important gene products activated by the Shh pathway (Figure 7) [23, 25, 26]. In this study, stimulation of BMSCs with NLTEs or ALITEs activated both the Shh and Wnt/*β*-catenin signaling pathways, as evidenced by the upregulation of the expression of the key proteins GLI1, Wnt3a, and *β*-catenin. However, the Shh pathway inhibitor GDC-0449 abolished these effects. Therefore, the Shh signal derived from the ALI lung microenvironment might drive the differentiation of BMSCs into AT2 cells through the activation of the Shh pathway and its downstream Wnt/*β*-catenin pathway in BMSCs. Given the in vitro experimental results, to further validate the role of the Shh pathway in the differentiation of BMSCs into AT2 cells, we transplanted GDC-pretreated BMSCs to treat LPS-induced ALI in mice. Notably, the capacity of the BMSCs pretreated with GDC to differentiate into AT2 cells was almost completely abolished in the injured lungs, as indicated by the severity and pathological injury score being similar to those of the LPS group.

In addition, unexpectedly, the number of engrafted BMSCs in the damaged lungs was significantly reduced in the GDC group, which might impair the viability or migratory capacity of the implanted BMSCs due to inhibition of the Shh pathway by GDC. The downstream target proteins of the Shh pathway are closely related to cell migration, survival, growth, proliferation, and differentiation (Figure 7) [25, 26, 39], and the Wnt protein and its related Wnt/*β*-catenin signaling pathway play crucial roles in the differentiation of BMSCs into AT2 cells [20]. Therefore, blockade of the Shh pathway in BMSCs not only suppressed Wnt protein expression, leading to a reduction in the differentiation efficiency of AT2 cells, but also inhibited their paracrine function by decreasing the expression of the antiapoptotic factor Bcl-2; the cell growth-related factors IGF2, FGF, and BMP; and the proliferation-related proteins Myc and cyclin D1 (Figure 6), ultimately resulting in reduced BMSC survival and inhibition of AT2 cell proliferation in the lung due to the reduction in growth factor secretion by BMSCs. Therefore, the transplantation of BMSCs inhibited by the Shh pathway in mice with ALI failed to alleviate lung injury; conversely, the regeneration of AT2 cells was impaired by reducing the efficiency of BMSC differentiation into AT2 cells and inhibiting AT2 cells proliferation (due to damage to BMSC paracrine function), ultimately weakening the therapeutic potential of BMSCs in the treatment of ALI/ARDS. Modulating the Shh pathway in transplanted BMSCs might be an effective strategy for improving the efficacy of BMSC therapy for ALI.

## 5. Conclusions

This study is the first to report that a relatively high proportion of BMSCs differentiate into AT2 cells in the early stage of treating ALI/ARDS with BMSCs, suggesting that the differentiation of BMSCs into AT2 cells is one of the important therapeutic mechanisms of BMSCs in the treatment of ALI/ARDS. The increased expression of Shh in injured lung tissue activated the Shh signaling pathway and its downstream key target protein Wnt3a during BMSC engraftment into the lung, thereby driving the differentiation of BMSCs into AT2 cells and ultimately promoting AT2 cell regeneration to repair injured alveolar epithelium and subsequently improve lung function in LPS-induced ALI/ARDS. Therefore, this study confirmed that activation of the Shh pathway in BMSCs significantly promoted the differentiation of BMSCs into AT2 cells in the damaged lung microenvironment. Enhancing the AT2 cell differentiation efficiency of BMSCs by modulating the Shh pathway might be an important strategy for improving the therapeutic efficacy of MSC treatment for ALI/ARDS.

## Data Availability

The data that support the findings of this study are available from the corresponding author upon reasonable request.

## Abbreviations

BMSC: Bone marrow mesenchymal stem cell
MSC: Mesenchymal stem cell
LPS: Lipopolysaccharides
AT2: Alveolar type Ⅱ
AT1: Alveolar type Ⅰ
ALI: Acute lung injury
ARDS: Acute respiratory distress syndrome
Shh: Sonic Hedgehog
H&E staining: Hematoxylin and eosin staining
ALITE: ALI lung tissue extract
NLTE: Normal lung tissue extract
SAGM: Small airway growth medium
SPC: Anti-prosurfactant protein C
T1*α*: Anti-podoplanin.
